# Painful Appendix Without Appendicitis: A Rare Presentation of Gut Malrotation

**DOI:** 10.7759/cureus.114188

**Published:** 2026-08-09

**Authors:** Muhammad Shoaib Anwar, Wardah Jabeen, Osama Shakeel, Abdelhamed Fanta, Menahil Noor, Nasir Z Ahmad

**Affiliations:** 1 General Surgery, University Hospital Limerick, Limerick, IRL; 2 Surgery, University Hospital Limerick, Limerick, IRL; 3 Colorectal Surgery, University Hospital Limerick, Limerick, IRL

**Keywords:** adult intestinal malrotation, appendectomy, appendix as hernial content, congenital intestinal anamolies, midline ceacum, supra umblical hernia

## Abstract

Intestinal malrotation is a rare congenital condition that occurs due to the developmental failure of mesenteric rotation. In adults, it can be either symptomatic or asymptomatic. Acute symptoms may include intestinal obstruction resulting from a volvulus, which can lead to perforation and sepsis. Chronic symptoms might consist of abdominal pain and loss of appetite, which can ultimately result in weight loss. In this report, we describe a case of an incarcerated ventral hernia in a patient who was incidentally diagnosed with malrotation through computed tomography (CT) imaging. The CT scan revealed that the cecum was located in the midline, with the duodenum positioned on the right side of the abdomen, a typical presentation for this condition. The CT scan further confirmed the presence of a ventral hernia containing an incarcerated omentum and a threatened loop of small bowel. However, the intraoperative findings were surprising. Instead of containing omentum or a loop of small bowel, the hernia contained the abnormally positioned appendix and cecum due to malrotation. The appendix appeared to mimic the small bowel, while the mesoappendix resembled the omentum, as seen on the CT scan. This case may be unique as it presents gut malrotation with an incarcerated and painful appendix within a ventral hernia.

## Introduction

Intestinal malrotation is a congenital condition that occurs when the intestines fail to complete an anti-clockwise rotation around the superior mesenteric artery during the 10th week of foetal development. This failure leads to issues with the positioning and fixation of both the small and large intestines [1]. The overall incidence of intestinal malrotation in live births is approximately 0.2%, with an estimated incidence of 0.2-0.5% in adults [2,3].

Nishjima classified intestinal malrotation into three different types: non-rotation, incomplete rotation, and incomplete fixation [1]. In adults, only 0.2-0.5% of these cases become symptomatic [2,4]. Typically, intestinal malrotation in adults is found incidentally [2,5]. In acute situations, malrotation can present as abdominal pain due to intestinal obstruction from Ladd's band or complications arising from volvulus [2-8]. Chronic presentations may be more subtle, consisting of non-specific abdominal pain, food intolerance, and bloating [2,3,6-8].

We present a case report of an adult woman who exhibited an unusual presentation of intestinal malrotation, manifesting as acute abdominal pain caused by an incarcerated ventral hernia in a supra-umbilical position.

## Case presentation

We present a case report of a 51-year-old female nursing home resident who attended the emergency department due to acute abdominal pain. The pain was primarily located in the central abdomen, accompanied by vomiting, loss of appetite, and altered bowel habits, including diarrhoea. Moreover, she reported swelling over the anterior abdominal wall for the past two weeks. Her medical history included a high body mass index (BMI) of 35.3 kg/m², a psychiatric disorder (bipolar disorder), and hypertension. Upon examination, the patient was hemodynamically stable, but her abdomen was extremely tender, with an irreducible lump in the supraumbilical region and no erythema.

Aside from grossly elevated inflammatory markers, her blood tests were largely normal. Neither an erect chest X-ray nor a plain film of the abdomen revealed any significant abnormalities. However, a computed tomography (CT) scan of the abdomen showed a narrow-neck ventral hernia containing loops of small bowel and indicated gut malrotation of the non-rotation subtype. The scan revealed that the position of the duodenum and small bowel was consistent with malrotation, with the duodenojejunal flexure located in the midline, small bowel loops on the right side of the abdomen, and large bowel on the left side, with the cecum centrally positioned.

After completing a thorough evaluation, we took the patient to the operating theatre for exploration of the hernia through an upper midline incision. Although we anticipated an incarcerated abdominal wall hernia preoperatively, the exploration of the hernial sac presented an unexpected finding: a macroscopically dilated appendix with a long mesentery and a portion of the cecum lying within the hernial sac. Further examination revealed that the cecum was located in the midline and was pulled into the hernial defect.

An appendectomy was performed through the midline incision, following standard protocol. We repaired the hernial defect without using synthetic mesh, employing interrupted polydioxanone sutures, and closed the skin with subcuticular Monocryl sutures.

**Figure 1:**
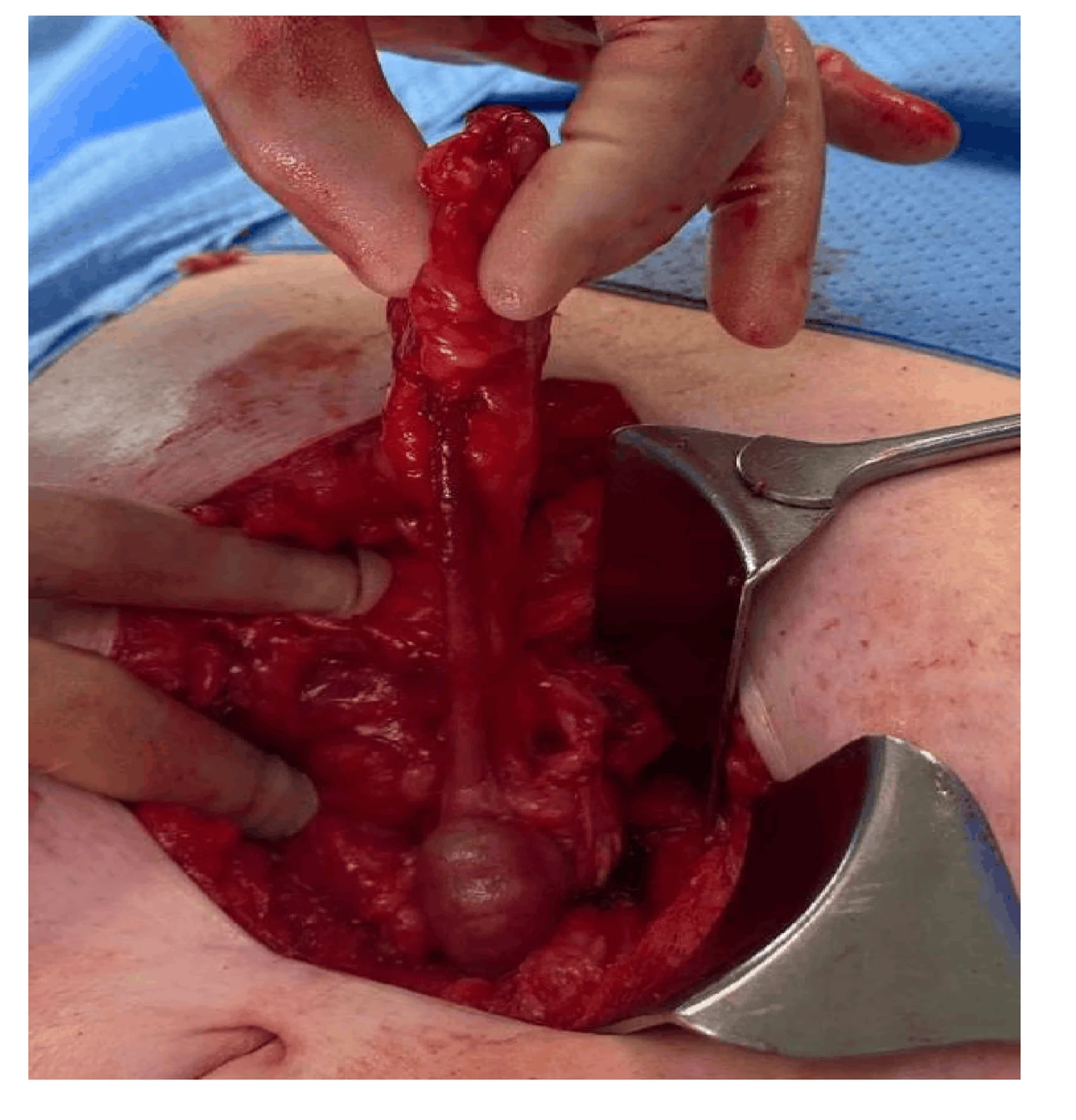
Operative ﬁnding of the appendix as a content of hernia

**Figure 2:**
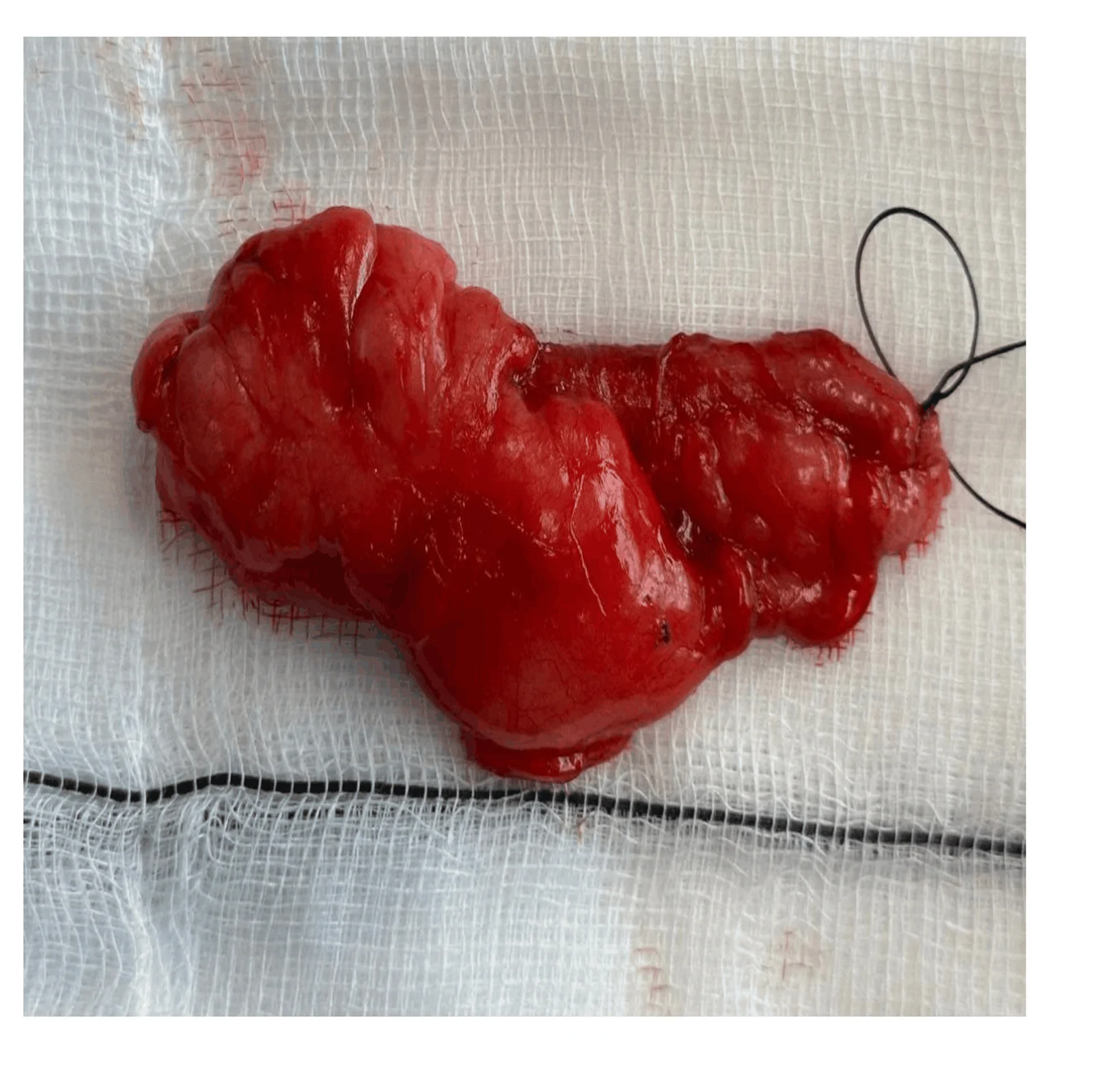
Appendiceal specimen

Her postoperative recovery was uneventful, and as anticipated, the histological analysis of the appendix showed no signs of inflammation. During a follow-up appointment three months later, there was no evidence of hernia recurrence or any postoperative complications. Figure 1 shows the appendix as the content of an umbilical hernia. Figure 2 illustrates the appendix specimen.

**Figure 3:**
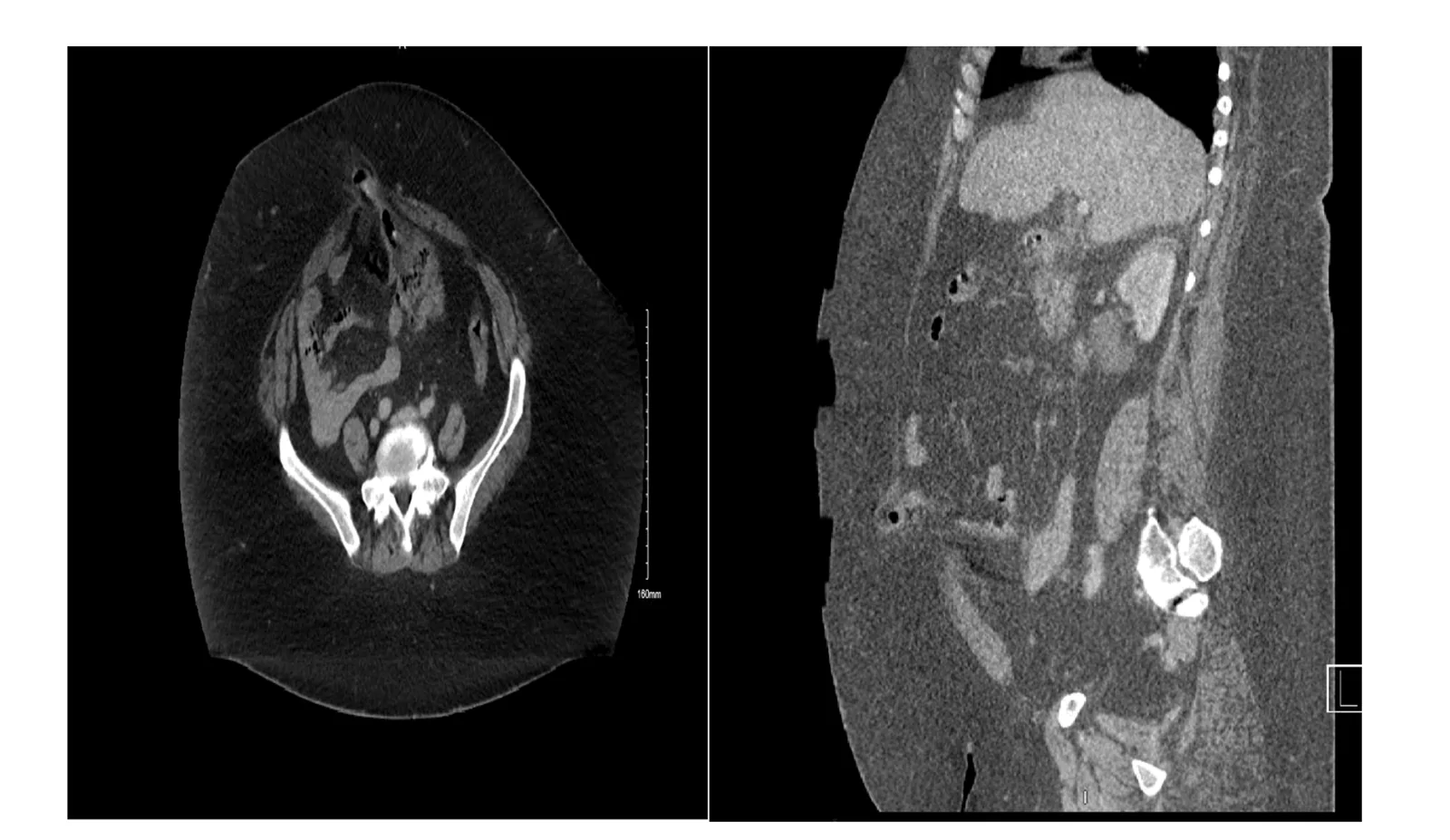
CT axial and sagittal sections showing paraumbilical hernia defect with its content

**Figure 4:**
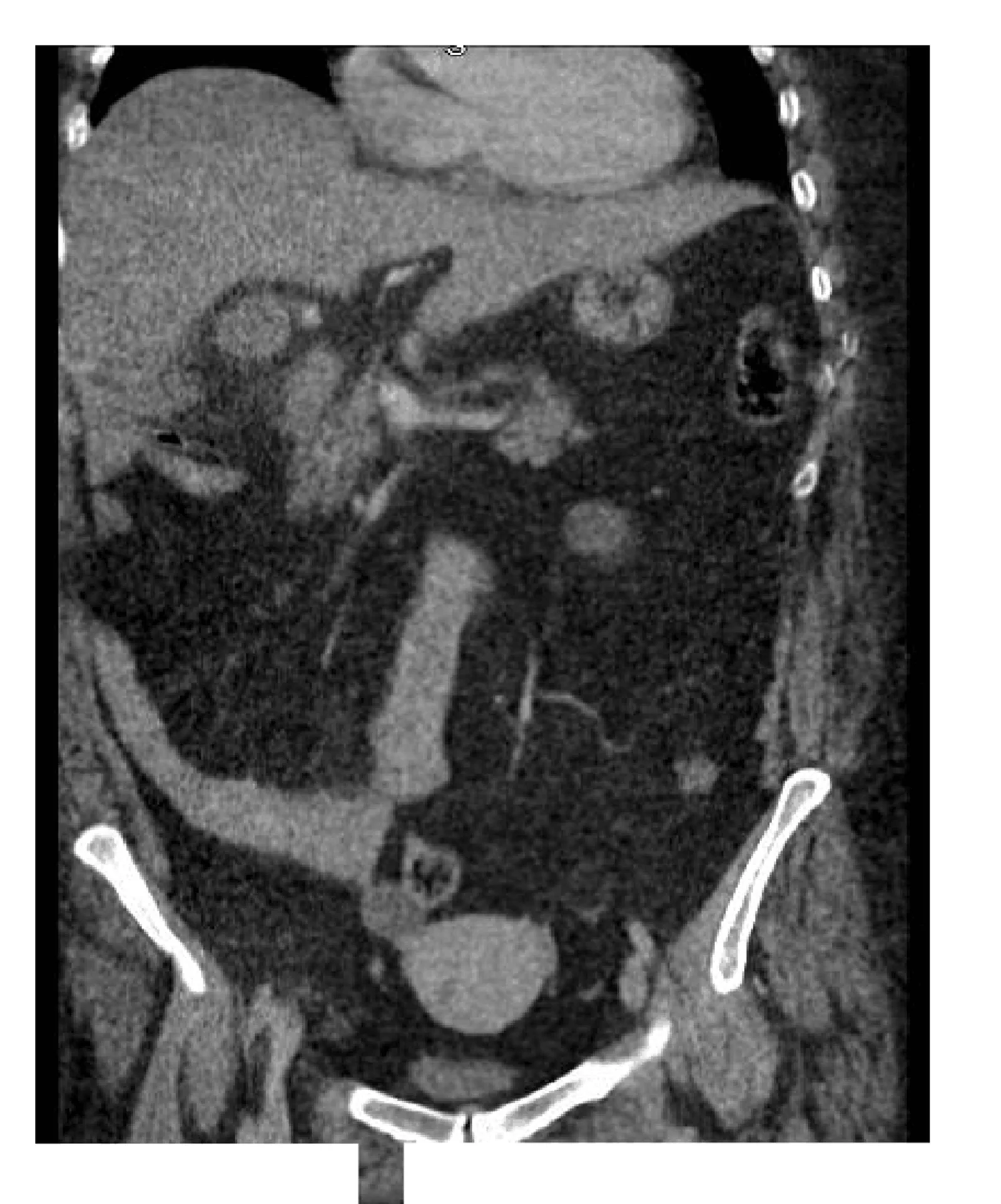
CT coronal section image showing the caecum in the midline and the small bowel on the right side

Figure 3 shows CT images of the axial and sagittal sections showing a para-umbilical hernia and its contents. Figure 4 shows the malrotation of the gut.

## Discussion

Malrotation of the gut is a developmental anomaly that can lead to acute or chronic symptoms later in life. In adults, the diagnosis is often incidental and discovered during imaging for another condition [2,5,7]. Malrotation is considered a contributing factor to many gastrointestinal symptoms [2,7,8]. Therefore, it is recommended to correct the anomaly to prevent future complications [5,7]. The surgical treatment for malrotation, known as Ladd’s procedure, involves fixing the mesentery in its correct anatomical position. This technique repositions the small and large intestines into their appropriate compartments [5,7]. 

A hernia is the protrusion of a viscus or part of a viscus through a defect in the cavity that contains it. Common types of abdominal wall hernias include inguinal, femoral, umbilical, paraumbilical, ventral, and incisional hernias [9,10]. In theory, any abdominal viscus can protrude through hernial defects in these locations. However, the organs or parts of organs directly beneath the defect are at a higher risk of herniation, which can lead to complications if not addressed in a timely manner [9,10]. In the case report described, the midline position of the caecum due to malrotation increased the risk of herniation through the defect of a ventral hernia. Prompt surgical intervention prevented strangulation of the appendix and caecum in the hernial defect, thereby averting further complications. Herniation of the appendix can occur in various locations, including right inguinal hernias, right femoral hernias, and umbilical hernias [11-17]. It is uncommon for the appendix to herniate on the opposite side of either an inguinal or femoral hernia [11,16]. In the published literature, there are nine case reports that discuss appendiceal herniation in a paraumbilical hernia [11]. The literature review indicated that nearly all patients presented with an irreducible and tender lump [11]. All patients underwent appendicectomy through the same incision for the paraumbilical hernia [11]. 

The standard treatment for hernias involving the appendix is an appendicectomy, regardless of whether the appendix is inflamed [11,12,14-17]. In our case, the appendix was dilated but did not appear inflamed, a finding confirmed histologically. The decision to leave the appendix intact in this scenario was likely not justified, as acute appendicitis in cases of gut malrotation could lead to diagnostic challenges and delays in management [5,7]. For this reason, corrective surgical procedures for gut malrotation usually include an appendicectomy [5,7]. 

This case report is noteworthy because it highlights two interrelated pathologies: gut malrotation with an abnormal position of abdominal viscera and the herniation of the caecum and appendix in a ventral hernia. It is advisable for clinicians to consider the possibility of other abnormalities in patients with malrotation, as unexpected findings can emerge [2,5,7]. 

## Conclusions

This case highlights an unusual and rare presentation of intestinal malrotation in an adult patient, manifesting as a paraumbilical hernia containing the appendix. The finding of the appendix as the sole content of the hernial sac is exceptionally uncommon and underscores the importance of considering anatomical variations during surgical exploration. Pre-operative imaging, particularly CT scanning, plays a crucial role in diagnosing both malrotation and hernial contents; however, intra-operative findings remain definitive. Prompt surgical intervention with appendicectomy and hernia repair led to a successful outcome. This report adds to the limited literature on such rare presentations and emphasizes the need for awareness among clinicians to prevent diagnostic and operative surprises.
